# Smart Protective Glove for Personal Protective Equipment (PPE) Against Chainsaws for Arborists

**DOI:** 10.3390/ma18051010

**Published:** 2025-02-25

**Authors:** Sandra Blocher, Dirk Wolff, Dennis Fassbender, Michael Schneider

**Affiliations:** 1 German Institutes of Textile and Fiber Research Denkendorf, Koerschtalstrasse 26, 73770 Denkendorf, Germany; 2 University of Applied Forest Sciences Rottenburg, Schadenweilerhof, 72108 Rottenburg am Neckar, Germany; wolff@institut-waldarbeit.de; 3 ALFA-ROTEC GmbH, Paul-Ruecker-Strasse 16, 47059 Duisburg, Germany; dennis.fassbender@alfa-rotec.de; 4 ATS Elektronik GmbH, Albert-Einstein-Strasse 3, 31515 Wunstorf, Germany; michael.schneider@atsonline.de

**Keywords:** chainsaw, personal protective equipment (PPE), occupational health and safety (OHS), arborist, tree care, forestry, clothing, glove, smart textiles, wearables

## Abstract

Working with a chainsaw is a risk for arborists. When handling a chainsaw, serious injuries can occur, particularly to the arms. For this reason, arborists must wear personal protective equipment (PPE) against chainsaws. This protective equipment corresponds to PPE category 3. Current protective gloves have several textile layers as protection. These gloves offer protection up to a chain speed of 24 m/s (class 2). No protective gloves for class 3 are available. A new approach is the solution presented in the paper, in which a smart glove and a modified electrical chainsaw can close this gap. For the development of the glove, the typical work situation, current accident situations, and accident statistics were analyzed. The legal requirements and standards for the European market and the wearing comfort are discussed; based on these data, gloves were designed that included electronics, and a chainsaw was configured accordingly. The glove was then tested under laboratory conditions to see whether the electronic functions in the glove could switch off the saw as soon as the glove came too close to it. The project showed the potential for smart textiles to overcome the limits in the layering of protective textiles.

## 1. Introduction

### 1.1. Accident Situation

In tree care, arborists use chainsaws. Work with chainsaws bears a risk of injuries. In 1994 in the United States, the use of chainsaws caused the most injuries to the arm and hand area and the leg area [1]. In Italy from 2007 until 2012, mainly the head, trunk, and legs were injured by accidents with chainsaws [2]. In 2018, 1757 accidents with arborists were reported to the German insurance company SVLFG. Sawing was the most common reason for injuries (785 cases). After branches and handheld saws, the chainsaw (185 cases) was in 3rd place on the list of objects and tools that caused such accidents. The main activity that causes an accident is getting hit by something; in second place is arborists cutting themselves (251 cases, including chainsaws and handsaws). The most dangerous work situation is sawing. The arborist either gets hit by branches or cuts themselves with a saw during the sawing activity. The most affected body part by chainsaws is the hand (414 of 1041 cases). Arborists often do not see their arms or hands because they are hidden by branches [3]. Based on accident statistics and scenarios, the requirements for protective gloves are defined below.

### 1.2. Gloves Used by Arborists

Functional principles of protective gloves: Current cut-protective gloves have several layers of cut-protective fabric sewn into the back of the hand. The cut protection is often a warp-knitted fabric made of chemical fibers that are pulled out of the glove when it comes into contact with the saw, blocking the saw. These gloves must meet the standard EN ISO 11393-4:2019 [4] when the chain is spinning out. In the standard are four different protective classes (class 0 at 16 m/s, class 1 at 20 m/s, class 2 at 24 m/s, and class 3 at 28 m/s) dependent on the speed of the chain when the saw comes in contact with the textile. All classes refer to a chain speed when no gas is given.

Current protective gloves: The gloves available on the market meet the standard up to cut protection class 2. Forest workers often work without class 2 gloves because of the limited comfort. Most glove models meet cut protection class 1.

Gloves worn by arborists without protection: Since arborists not only have to work with saws but have to climb in the trees, they often prefer thin gloves that allow a secure grip when climbing in the trees. Thick, multilayered gloves are particularly disadvantageous for arborists. During work, the fingers should be protected by a glove, but thick gloves can hinder the secure handling of the chainsaw [5]. For this reason, arborists often use only thin, knitted gloves that have a nonslip coating on the palm. These knitted gloves do not provide any protection against the chainsaw.

Lack of comfort and limitations of protection: All personal protective equipment (PPE) for chainsaws currently includes several layers of cut-protection textile. This protective textile is in gloves only on the top of the fingers and hands. In order to achieve cut protection class 3 with the current textile cut protection, more than 7–10 layers of textile are used in cut-protection trousers. For a glove, a 10-layer construction would mean the loss of tactility and freedom of movement. Since the cut-protective gloves available on the market protect only up to a maximum of protection class 2, there exists a market gap for cut protection class 3. At full throttle, a chainsaw can reach speeds of up to 35 m/s. Currently, no glove on the market can stop a chainsaw at full speed.

### 1.3. Current Trends in PPE and Chainsaws for Smart Personal Protective Systems (SPPS) in Forestry

Development of electrical chainsaws: Especially for arborists, there is a great risk that the saw will come into contact with their fingers. Branches are often lifted with one hand while the saw is operated with the other hand. The further development of top-handle saws (motorized tree-care saws) also means that arborists who have the appropriate training are increasingly operating chainsaws with just one hand. Because of the high risk potential of this type of saw, the manufacturers point out that top-handle saws must not be operated with one hand and may only be used as intended for tree care.

Further development of smart PPE: Research has made progress in personal protective equipment by combining textiles and new technologies. Smart textiles can improve the protective effect of PPE [6]. By extending PPE with smart functions, protection of the wearer at full chain speed could be achieved. Marchal et al. call this new type of PPE “Smart Personal Protective Systems (SPPS)” [7]. With SPPS, the protection and comfort level can be higher than in state-of-the-art solutions [8]. In a research project, Beringer et al. developed chainsaw-protective trousers with sensors [9].

### 1.4. Aim of the Work

The aim of the work is to develop a glove which contains smart protective elements and which is accepted by the arborists at work. The purpose of this work is to elaborate a concept for future smart gloves for arborists. The focus is to create an active PPE solution. The advantages of active PPE as a protection against chainsaws are listed in Table 1 below. Compared to [9] in this work a real electrical chainsaw is modified to interact with the glove. The glove and the machine shall interact as a protective system. 

## 2. Materials and Methods

For the development of the glove, accident statistics and accident situations were analyzed. For the correct selection of textiles and the pattern of the glove, various classic work situations for arborists were discussed and categorized. In addition, tree care professionals were asked how they imagined a smart protective glove. The advice of the arborists was incorporated into the design of the glove. As the glove was to belong to PPE class 3, the legal and normative requirements for such a product also had to be considered. Since the glove was designed for the European market, regulations and standards that are valid in the EU for placing class 3 PPE on the market were reviewed, and the relevant requirements that specifically concerned the product were listed and transferred into specifications. Based on the requirements listed above, several gloves were produced. The prototypes were discussed with the project team and with arborists. When evaluating the gloves, three aspects in particular were important: fulfillment of the legal aspects, wearing and working comfort for the user, and the durability and reliability of the integrated electronics. At the end, a final prototype was tested together with a modified electrical chainsaw in a laboratory environment. During the test, particular attention was paid to ensuring that the saw stopped in time if it came too close to the glove.

## 3. Results

### 3.1. Regulations and Standards for Protective Clothing Against Chainsaws

To be allowed for sale on the European market, a smart protective glove must comply with REGULATION (EU) 2016/425 [10]. To be reliable products, the gloves must meet certain requirements and therefore undergo testing procedures. PPE must provide protection against the hazards it is intended to protect against. The sensory protective glove for arborists is intended to protect against chainsaw cuts. In addition, the PPE regulation stipulates that other risks that may arise during the intended use must be considered. For this purpose, a risk analysis of work situations on trees was carried out. It emerged that mechanical risks exist in every work situation. Therefore, protection against mechanical risks and protection against chainsaw cuts were defined as basic requirements for the protective effect of the sensory protective glove. The general requirements for protective clothing are described in DIN EN ISO 13688:2022-04 [11]. This standard is relevant for general ergonomic aspects, the safety of the material, and the kind of information the user should receive about the PPE. In this work, the standard DIN EN ISO 11393-4 [4] was therefore considered important for protective gloves intended for users of handheld chainsaws, as this standard describes the requirements for protective gloves against chainsaw cuts. The most basic standard for gloves is DIN EN ISO 21420 [12], which deals with general requirements and testing procedures for protective gloves. Because of the variable areas of application of protective gloves, it is necessary to use standards that relate to specific areas of application. This standard in turn refers to the standard DIN EN ISO 388:2019-03 [13], which deals with protective gloves against mechanical risks.

Therefore, protection against mechanical risks and protection against chainsaw cuts were defined as basic requirements for the protective effect of the sensory protective glove. The standards and examples for the development of an arborist-specific product are listed in Table 2.

### 3.2. Additional Regulations and Standards Due to the Smart Function in the Glove

The developed glove can be classified as smart PPE with electronics according to the classification in [8], and it interacts with a machine. Therefore, regulations and standards from the area of electronical products and machine construction are also relevant. The glove contains electronics, which should be safe to use. The LVD (2014/35/EU) [14] sets out essential safety requirements to protect users from electric shock and other hazards. For the interaction between the saw and the glove, it is important that the system is not disturbed by electromagnetic inference. The requirements on electromagnetic compatibility are listed in EMC Directive (2014/30/EU) [15]. In order to comply with the RoHS Directive (2011/65/EU) [16], the electronic components must not contain any harmful substances or substances prohibited in the EU. Because of the interaction between the glove and the saw, the glove can be seen as an additional part of a machine. Therefore, the Machinery Directive (2006/42/EC) [17] was considered to guarantee the safety of the interacting system. Table 3 shows the standards mentioned above with specific examples for the arborist’s glove.

### 3.3. Different Work Situations and the Risks They Present of Getting Injured by the Chainsaw

Together with the project team and an instructor for arborists, the typical work situations of arborists were identified. The work situations can be divided into three categories (Table 4): working with the ropes, performing tree care in the tree, and tidying up.

### 3.4. Requirements on the Design and Pattern of the Glove Dependent on the Work Situation

For the development of the glove, the work of arborists was analyzed and discussed with arborists and tree care instructors. During the work analysis, attention was paid to which objects and surfaces the glove would come into contact with and which hand movements the arborist would perform.

Requirements when working with the rope: Since the arborist’s hands grip, hold, pull, and tie the ropes, there is a large amount of friction on the insides of the hands and fingers. The ropes also have a rough surface. It was therefore concluded that the protective gloves should have sufficient protection against mechanical injuries, such as high abrasion resistance and tear resistance, on the insides of the hands and fingers. In addition, the fingers need to have high levels of tactility, mobility, and grip so that climbing tools can be operated and ropes can be gripped and tied tightly. Therefore, the protective gloves should allow for a high level of finger mobility and have high levels of grip and tactile sensitivity at the fingertips. In addition, fingers can get caught between ropes or in climbing tools such as carabiners or ropes when tying knots, which can lead to crush injuries. When the arborists were questioned, it emerged that it was important that the glove should have no tabs, buckles, or raised areas and that it should fit very tightly so that nothing could get pulled into safety devices when working with the rope and that the rope is not blocked as a result.

Requirements when the arborist climbs: The rough and sharp-edged surfaces of trees can lead to cuts and abrasions. It was therefore deduced that the protective gloves should offer protection against mechanical injuries, such as high abrasion resistance, cut resistance, and tear resistance. The German Social Accident Insurance (DGUV) also describes the risk that the hands can come into contact with plant parts, splinters, spines, and thorns, which can penetrate the skin and cause inflammation or allergies [18]. Furthermore, dirt and small branches, if they get into the gloves, can disturb the arborists at work. Dirt with a certain amount of residual moisture on the gloves can also impair the sensitivity and grip of the hand, as well as the functionality of the sensors and electronics. It was therefore deduced that the protective gloves and the electronics should be resistant to small branches and dirt getting in.

Requirements when the arborist saws: There is a high risk of cuts when sawing with a chainsaw, as the arborist can saw into their free hand when operating the chainsaw with one hand. Branches and leaves can increase the risk of cuts if they restrict the view of the hands. The arborist could also injure themselves with the chainsaw by slipping from the working position. There is also a risk of injury from escaping substances such as petrol or oil if the hand slips, as these can make the handle of the saw slippery. There is a risk of cuts for both hands. Splinters of wood can be thrown into the air when sawing, which can cause injuries if they hit the body. Finally, the DGUV sees frequent hazards in one-handed operation of the chainsaw, sawing in unsafe positions, kickback of the chainsaw, and flying chips, splinters, and fuel [19]. From this, requirements for protective gloves were derived, such as very high protection against chainsaw cuts on both hands, very high grip on the palms of the hands, and impermeability to wood splinters. The electronic cut protection must provide complete cut protection on all fingers of both hands and arms. In addition, the protective gloves should be water and oil repellent and clearly visible. The integrated electronics and sensors should also be protected from water and oil.

Requirements when the arborist tidies up: Rough and sharp-edged surfaces of trees can lead to cuts and abrasions. Therefore, the protective gloves should offer protection against mechanical injuries such as high abrasion resistance, cut resistance, and tear resistance. It is important that the glove has a tight hem to avoid dirt or small branches entering inside of the glove.

The requirements dependent on the work situation are listed in the Table 5 below.

### 3.5. Different Patterns and Designs for the Glove

According to the requirements listed in Section 3.1, Section 3.2, Section 3.3 and Section 3.4, several glove patterns were created. At the beginning, four gloves were made without sensors in order to find the right fit for the intended use.

**Figure 1:**
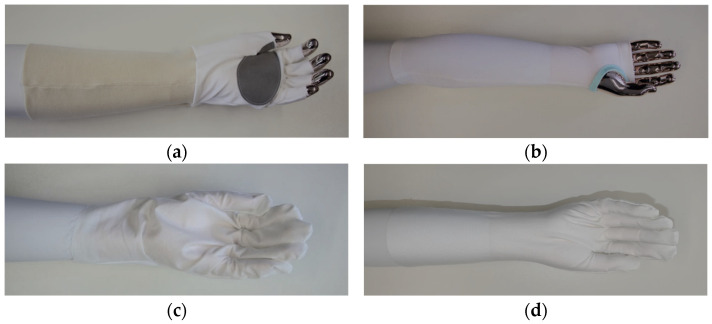
Four glove models: (**a**) climbing glove; (**b**) underarm cuff; (**c**) five-finger glove with nonelastic fabric; (**d**) five-finger glove with elastic fabric only.

Climbing glove: This glove model, which is shown in Figure 1a, was inspired by a traditional climbing glove because arborists climb often during their work. To achieve good tactility for ropes and tree care, the glove had open fingers. For better movement and fit, the glove was made with elastic fabric (white fabric in (a)). The cuff of the glove reached the elbow to achieve cut protection with sensors for the underarm. For climbing with ropes, the palm was reinforced with a nonelastic durable fabric (dark grey fabric in (a)). This glove model consisted of several different fabrics and pattern parts. Therefore, this glove was time consuming to sew.

Underarm cuff: This model, shown in Figure 1b, consisted of one pattern part and can be sewn very quickly. The knitted elastic fabric allows the arborist a high freedom of movement. The fingers are open, and the thumb hole prevents the cuff from sliding up. The idea of this model was that the sensors could be integrated at the underarm and that the arborists could choose and change their preferred gloves for protection against dirt and scratches.

Five-finger glove with nonelastic fabric: The five-finger glove shown in Figure 1c consisted of two identical pattern parts. This glove can be sewn with an automated sewing machine or embroidery machine. The back of the hand was made with an elastic fabric to enable enough freedom of movement when the hand is bent. The nonelastic fabric was at the palm of the hand and could be used to embroider sensors on the fabric.

Five-finger glove with elastic fabric only: The five-finger glove shown in Figure 1d had two identical pattern parts and can be sewn automatically. Both sides of the glove were made out of elastic fabric. Therefore, this glove had more freedom of movement and could fit to more persons. The disadvantage is that sensors and conductive paths could not be applied on the fabric as easily as on nonelastic fabrics.

### 3.6. Asking Arborists About the Four Glove Models and Their Wearing Behavior of Gloves

The gloves listed in Section 3.5 were discussed with arborists and industry partners. Four glove designs without sensors were sewn to ask arborists about their preferred glove design. The results for each glove model were as follows.

Climbing glove: The climbing glove was not preferred by any arborist because of the open fingers. During the work, dirt, dust, small branches, and needles could accumulate in the glove.

Underarm cuff: The arborists thought that this glove was suitable to wear. One major advantage for them was that they could still wear their personally preferred working gloves above the smart protective underarm cuff.

Five-finger glove with nonelastic fabric: The nonelastic fabric caused too many pleats when the hand grabbed something. The arborists were concerned that fabric could be pulled into rope safety devices while working with ropes.

Five-finger glove with elastic fabric only: All arborists liked this model because of the high flexibility and tactility. Arborists expressed concerns about when the glove would be equipped with smart functions. The glove would only last a few days because of the highly abrasive work, and it would be expensive to throw away the dirty and broken glove with the electronics.

Wearing behavior: It turned out that arborists preferred very individually different gloves. Gloves with protection against chainsaws were not worn, as arborists and forest workers prefer a variety of different gloves and change them several times a day depending on the operation. As a result of the discussion, it was decided to continue the development with the arm cuff and a flexible five-finger glove. The cuff has the advantage that arborists can still wear their own gloves and change them individually dependent on the current work situation. Another advantage is that the electronics of the cuff can be protected by the glove worn above. The cheap working gloves can be thrown away when they are broken while the smart cuff remains. An extended underarm cuff could enlarge the cut-protected area and could be used for the integration of electronics.

### 3.7. Asking Arborists About the Preferred Position of the Electronics

**Figure 2:**
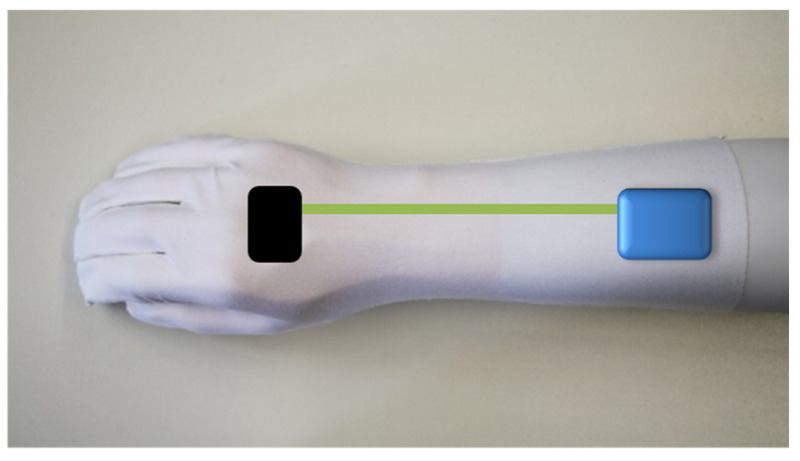
Position of the electronic parts on the glove. The sensor (black) at the back of the hand was connected via conductive tracks (green) with the battery and Bluetooth module (blue box).

In addition to the design of the gloves, the arborists were also asked where the electronics on the gloves should best be placed. Since the battery and Bluetooth modules would be housed in a solid housing, this component would not be deformable and would protrude beyond the glove. Since this protrusion would represent a potential risk when working with the rope, it was decided to attach the solid housing to the forearm, as shown in Figure 2. The connection between the sensors on the wrist and the electronics housing was made via conductor tracks.

### 3.8. Integrating Sensors and Electronics into the Arm Cuff

**Figure 3:**
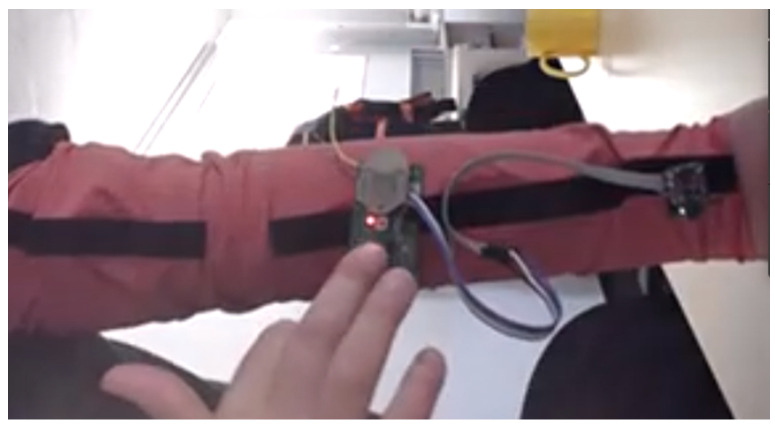
Elastic arm cuff equipped with electronics.

**Figure 4:**
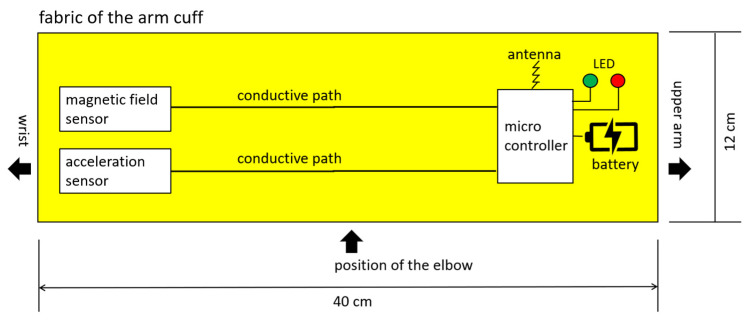
Scheme of the protective arm cuff with the positions of the electronic parts. The yellow square is the fabric of the arm cuff.

As a result of the discussion with arborists, an elastic arm cuff was equipped with electronics, shown in Figure 3. To achieve an interaction between the arm cuff and the electrical chainsaw, the cuff contained the electronic parts listed in Table 6. The placement of the electrical parts in the cuff is shown in Figure 4.

### 3.9. Testing the Arm Cuff Under Laboratory Conditions

**Figure 5:**
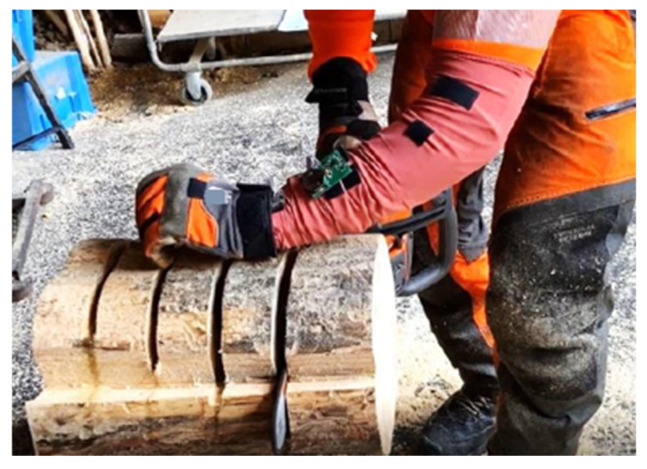
Testing the sensitivity of the system with different cutting depths in a tree trunk.

**Figure 6:**
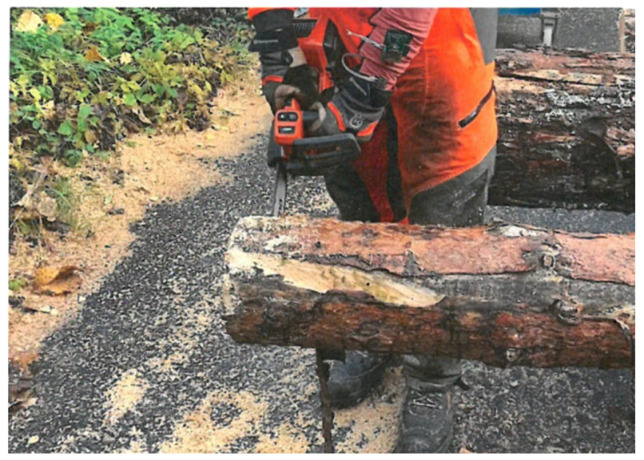
Testing the system by cutting a tree trunk.

To validate the concept of the protective function, the underarm cuff was tested together with a modified electrical chainsaw. The aim of the test was to observe the interaction between the arm cuff and the chainsaw. The arm cuff was worn by one test person on the left arm. After a calibration of the system, the test person held the modified electrical chainsaw in the hand. To see whether the saw and the glove interacted and were coupled with each other, LEDs were attached to the saw and the arm cuff. If the LEDs were green, both products were connected to each other during the work situations, and the interaction behavior was analyzed. The system was tested in open air. At the beginning of the test, the sensitivity of the sensors was measured at different distances and angles. The chainsaw approached the arm cuff from different directions, under different angles, and with different velocities to simulate what could happen when a user operated the chainsaw. Various movements were investigated under usage conditions for release behavior. The chainsaw stopped between 2.5 ms and 9.8 ms. This helped to determine the positioning of the sensors in the textile. In addition, detection tests were carried out with a tree trunk at different cutting depths, as shown in Figure 5. Typical work situations such as cutting tree trunks were tested, as shown in Figure 6.

Two important findings were to determine the sampling rate required for the application and the sensitivity. The positioning of the sensors could be determined. The result of the test was that the system interacted well. The chainsaw stopped in a timely manner when the arm cuff came too close.

### 3.10. Final Arm Cuff

**Figure 7:**
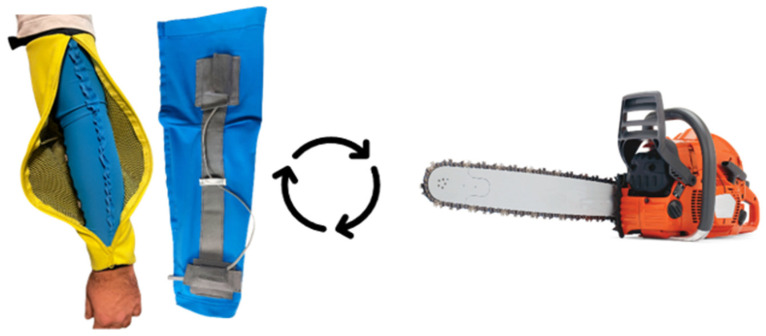
Final protective arm cuff, with sensors, battery, and additional protective yellow fabric, which was able to interact with a modified electrical chainsaw.

After the tests, a final arm cuff was made. This arm cuff had an additional protective layer, yellow fabric in Figure 7. The yellow fabric was protection for the conductive paths and the sensors. The cuff could be opened by a zipper. The zipper allowed the operator to change the battery and control the electronics optically.

## 4. Discussion

### 4.1. Design and Electronics

The authors agree with Buchweiller et al. that electronics and circuits that are part of PPE should have at least the same protection reliability as traditional PPE. New risks should not exist, and there must be confidence in new smart PPE [20]. The sensors and electronics that are responsible for the main safety function must have a defined accuracy. The accuracy of the electronics and sensors used for the protective arm cuff should be tested in further tests. For example, it is important for the end user to have an indicator light that shows that the system is working. This would allow arborists to rely on the system because they can see that the saw and the glove are connected.

### 4.2. Test Area and Number of Tests

The functional tests were carried out on a small scale. The final arm cuff was tested together with the configured electrical saw. The tests took place in a laboratory environment. For further developments and research, it is desirable to have functional tests take place in real-world working conditions. More data about the system’s performance must be collected under environmental influences such as different weather conditions (humidity, extreme cold, or extreme heat), vibration, strong electromagnetic interference conditions, and dirt. Human influences such as sweat and washability must be further analyzed. Tree care is physically demanding work, so workers sweat a lot. This causes the smart PPE to become damp. Both the moisture and the salts contained in sweat can affect the output values of the sensors. It is therefore advisable to test the smart PPE when sweaty. Storing the sweaty PPE can also have an impact on the sensors and electronic parts, as the conductive salts in the sweat dry out during storage and are deposited on the sensors when worn several times. To obtain more information about the reliability of the system, the safety functions of the glove and the saw should be tested over a longer period of time and in different work situations. Above all, the reliability of the electronics must be checked. The saw’s shutdown function should work in every work situation so that arborists do not feel hindered in their work by the smart functions.

### 4.3. Missing Standards and Test Methods

A reliable test environment should be created not only in a real work situation but under laboratory conditions, especially for notified bodies. The new protective functions must be developed with high reliability and tested properly. New test methods and standards for smart PPE must be elaborated. Reliability must be tested not only for the individual components of the system but for the entire system. Furthermore, the acceptance of users is important for the success of such new technologies at work. Therefore, the users must be well informed [8]. The authors agree with the current and foreseeable problems of smart PPE listed by Dolez et al. in [21]. To move forward in the area of smart PPE, more specific standards and guidelines are needed. Companies that start to produce smart PPE need guidelines to design and develop it. Notified bodies need standards and test methods to prove the conformity of smart PPE to regulations.

The development of textile-based smart PPE involves a systematic approach to ensure safety, functionality, and reliability. Companies engaged in this field should begin by selecting electronic parts and textiles based on the required accuracy, relying on data sheets. Once the components are selected, preliminary testing should be conducted to assess the interaction between the electronic components and textiles under controlled laboratory conditions, which is crucial for identifying any potential issues early in the development process. After preliminary tests, the next step could be to test the fully assembled system in the laboratory, evaluating the overall functionality and integration of the components. To ensure robustness, the system should then undergo testing under extreme laboratory conditions (temperature, humidity, and other environmental factors). The final testing phase could involve deploying the system in the actual work environment where it will be used, which is essential for observing how the smart PPE performs under real-world conditions and gathering feedback for further improvements and obtaining early feedback from users.

To ensure compliance and performance, notified bodies play a significant role in the testing and certification of smart PPE. They conduct type testing to evaluate the performance of smart PPE against established standards, ensuring it meets safety and operational requirements. Additionally, notified bodies perform quality management system audits of the manufacturing processes to ensure alignment with regulatory standards, helping maintain quality throughout production. Beyond laboratory assessments, they may also conduct field testing to observe the system’s performance in practical applications.

### 4.4. User Acceptance

New protective technologies can be successful only when users like to use them, in this case to wear the smart PPE against chainsaws. In the Table 7 below, concerns users might have are listed along with methods how those concerns could be reduced.

## 5. Conclusions

Protection against chainsaws by textiles is often limited by the thickness and lack of comfort of those textiles. Smart textiles can overcome this limitation by creating lightweight protective solutions. New concepts of textile-based smart protection systems can offer better means of protection. The arm cuff shows how accidents can be avoided in future by connectivity and interaction between machines and operators. In the project, it turned out that arborists had difficulty accepting a smart glove with fingers because they want to wear and choose their own gloves. An intelligent arm cuff was the solution. The functional tests showed that an interaction between the arm cuff and the saw worked. The saw was stopped as soon as the worker’s arm came too close to the saw.

## Figures and Tables

**Table 1:** Advantages of active PPE as a protection against chainsaws.

	Passive Protection	Active Protection
Dangerous situation	Protects when danger occurs	Protects before danger occurs
Activation of the protective function	The chainsaw is already too close to the body. The chainsaw touches the fabric and stops when the fabric is destroyed.	The system is activated when the chainsaw is close to the body. The chainsaw stops before the saw touches the fabric of the PPE.
Protection principle	Mechanical protection	Electrical protection
Example	Existing textile-based PPE Protection against chainsaws: several layers of cut-protective fabric	Smart personal protective systems (SPPS) Protection against chainsaws: smart protective glove for PPE
Wearing comfort	thick -> very warm -> sweating -> mobility restrictions	thin -> less sweating -> higher tactility

**Table 2:** Regulations and standards for chainsaw-protective gloves in Europe and examples for the development herein.

Standard	Examples Concerning a Smart Protective Glove for Arborists
REGULATION (EU) 2016/425 On personal protective equipment [10]	- the new technology should not be an additional risk - no danger due to the shape, surface, or material - the design must prevent pinching or getting caught in branches and sawing elements - no protruding elements such as tabs, buttons, loops, etc.
DIN EN ISO 13688:2022-04 Protective Clothing—General Requirements [11]	- must not slip despite the worker’s movement - must not hinder movement - the glove should be able to adjust to the size of the hand - no use of rough or sharp-edged materials - easy to put on and take off - must not prevent other important PPE items from being worn
DIN EN ISO 11393-4 Protective Clothing for Users of Hand-Held Chainsaws [4]	- highest protection against chainsaw cuts on all fingers of both hands in protection class 3 (chain speed of (28 ± 0.2) m/s)
DIN EN ISO 21420 Protective Gloves—General Requirements [12]	- no harmful materials in the textile and electronics - the foreseeable period of use must be labelled
DIN EN 388:2019-03 Protective Gloves Against Mechanical Risks [13]	- protection against mechanical risks - high abrasion resistance against rough surfaces (at least performance level 2) - high tear resistance (at least performance level 2), high cut resistance against sharp-edged surfaces (at least performance level 1 or A) - high puncture resistance against sharp plant parts (at least performance level 2)

**Table 3:** Additional regulations and standards due to the smart functions in the glove and examples for the development.

Number	Examples Related to the Glove
Low Voltage Directive 2014/35/EU	- protection of electronics and sensors from moisture, sweat, dirt, petrol and oil - no binding of sweat or water when the glove is wet
EMC Directive 2014/30/EU	- the glove does not emit electromagnetic inference - the smart function of the glove does not disturb other electrical equipment close to the glove
Machinery Directive 2006/42/EC	- the interaction between the glove and the saw must be safe and reliable
RoHS Directive 2011/65/EC	- no hazardous substances in electrical and electronic equipment - no use of lead-containing solder - when metal parts come into contact with sweat, no harmful substances may be produced

**Table 4:** Description of different work situations and their risk of getting injured by the chainsaw.

	Work Situation	Description
Working with ropes and climbing	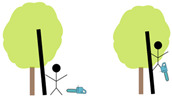	The arborist attaches or detaches the rope or climbs Risk of injuries by the saw: low when the chain brake is engaged or the saw is turned off; sharp chain
Sawing	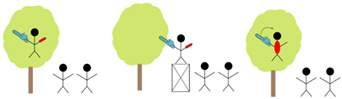	The arborist saws in the tree or on a platform Risk of injuries: holding the chainsaw with one hand and cutting into the other hand; getting a kickback into the body; sawing but not seeing body parts and cutting into the body
Tidying up	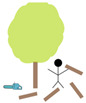	The arborist cleans up Risk of injuries: holding the chainsaw with one hand and cutting into the other hand; getting a kickback into the body

**Table 5:** Construction requirements for the glove dependent on the work situation.

	To Attach Ropes/ to Rope Down	To Climb	To Saw	To Tidy Up
Tactility	High	High	Low	Middle
Covered fingers	All fingers must be covered by a glove	All fingers must be covered by a glove	All fingers must be covered by a glove	All fingers must be covered by a glove
Fit	Tight	Tight	Tight hem to avoid dirt inside of the glove	Loose or tight fit
Fabric	Thin, good grip, robust, resistant to abrasion, moisture and dirt repellent	Thin, good grip, robust, resistant to abrasion, moisture and dirt repellent	Good grip, signal color (left), robust	Robust, moisture and dirt repellent, resistant to abrasion
Sensors	Active	Active	Active	Active
Influences	Moisture, mud, abrasive surfaces	Moisture, mud, abrasive surfaces, sharp edges	Oil, dust, petrol	Moisture, mud, abrasive surface, sharp edges

**Table 6:** The electronic parts used and their functions in the arm cuff.

Electronic Parts	Function of the Electronic Parts
Bluetooth low-energy module	Detection of persons, communication with the saw
Magnetic field sensor	Detection of the chainsaw
IMU (inertial measurement unit)	Detection of kickbacks and movements
Battery	Energy source
Red and green LEDs	Showing that the system is active and works well (green light) or has a malfunction (red light)
Microcontroller	Configuration and control of the peripheral areas

**Table 7:** Concerns of users about smart PPE and how they could be reduced.

Concerns of the User	How the Concerns Can be Reduced
Reliability: Users may worry about the reliability of the new technology.	- testing under various conditions by notified bodies - certifications that demonstrate reliability and performance - explaining and demonstrating the new technology in detail - combining with old/traditional protective textile technology at the beginning - calibrating and checking the system before use - red/green LEDs to show that the system is working properly
Cost: Smart PPE can be more expensive than traditional PPE.	- highlighting reduced injury rates - mentioning lower healthcare costs and increased productivity - providing case studies demonstrating cost effectiveness over time
Complexity: Users may find smart PPE complicated to use.	- the operation of the protective equipment should be as simple as possible - the design and handling should be self-explanatory - offering comprehensive (online) training sessions - providing user-friendly manuals - ongoing support to help users feel confident in adopting smart PPE
Comfort: Users may find smart PPE uncomfortable to wear.	- ergonomic and lightweight design - placing stiff and hard electronic components in the right places
Usability: Users may find smart PPE uncomfortable to use.	- the protective function should not hinder the workflow - getting feedback from users during the development process to improve design and functionality - early usability tests during the development process
Durability: Users may worry about the durability of the technology, especially in harsh environments.	- rigorous testing under various conditions - certifications that demonstrate durability and performance - establishing a test standard for smart PPE - establishing new smart textile test methods for textile-based smart PPE
Data Privacy and Security: Users may be concerned about how their data are collected, stored, and used.	- clearly communicating data privacy policies and ensuring compliance with regulations - offering transparency about data usage and providing options for data control
Maintenance and Support: Users may be apprehensive about the maintenance required for smart PPE.	- offering robust customer support and maintenance services, as well as easy-to-follow maintenance guidelines - providing warranties or service agreements to assure users of ongoing support

## Data Availability

The datasets presented in this article are not readily available because the data are part of an ongoing study. Requests to access the datasets should be directed to the authors.
